# Transforming Pharmacogenomics and CRISPR Gene Editing with the Power of Artificial Intelligence for Precision Medicine

**DOI:** 10.3390/pharmaceutics17050555

**Published:** 2025-04-24

**Authors:** Amit Kumar Srivastav, Manoj Kumar Mishra, James W. Lillard, Rajesh Singh

**Affiliations:** 1 Department of Microbiology, Biochemistry, and Immunology, Morehouse School of Medicine, 720 Westview Drive SW, Atlanta, GA 30310, USA; asrivastav@msm.edu (A.K.S.); jlillard@msm.edu (J.W.L.J.); 2 Department of Biological Sciences, Alabama State University, Montgomery, AL 36104, USA; mmishra@alasu.edu; 3 Cancer Health Equity Institute, Morehouse School of Medicine, 720 Westview Drive SW, Atlanta, GA 30310-1495, USA

**Keywords:** pharmacogenomics, CRISPR gene editing, artificial intelligence, precision medicine, genetic biomarkers

## Abstract

**Background**: Advancements in pharmacogenomics, artificial intelligence (AI), and CRISPR gene-editing technology are revolutionizing precision medicine by enabling highly individualized therapeutic strategies. Artificial intelligence-driven computational techniques improve biomarker discovery and drug optimization while pharmacogenomics helps to identify genetic polymorphisms affecting medicine metabolism, efficacy, and toxicity. Genetically editing based on CRISPR presents a precise method for changing gene expression and repairing damaging mutations. This review explores the convergence of these three fields to enhance improved precision medicine. **Method**: A methodical study of the current literature was performed on the effects of pharmacogenomics on drug response variability, artificial intelligence, and CRISPR in predictive modeling and gene-editing applications. **Results**: Driven by artificial intelligence, pharmacogenomics allows clinicians to classify patients and select the appropriate medications depending on their DNA profiles. This reduces the side effect risk and increases the therapeutic efficacy. Precision genetic modifications made feasible by CRISPR technology improve therapy outcomes in oncology, metabolic illnesses, neurological diseases, and other fields. The integration of artificial intelligence streamlines genome-editing applications, lowers off-target effects, and increases CRISPR specificity. Notwithstanding these advances, issues including computational biases, moral dilemmas, and legal constraints still arise. **Conclusions**: The synergy of artificial intelligence, pharmacogenomics, and CRISPR alters precision medicine by letting customized therapeutic interventions. Clinically translating, however, hinges on resolving data privacy concerns, assuring equitable access, and strengthening legal systems. Future research should focus on refining CRISPR gene-editing technologies, enhancing AI-driven pharmacogenomics, and developing moral guidelines for applying these tools in individualized medicine going forward.

## 1. Introduction

Precision medicine represents a paradigm shift from traditional uniform treatment approaches to highly individualized therapeutic strategies tailored to an individual’s genetic, environmental, and lifestyle factors. This evolving discipline integrates advancements in genomics, molecular biology, and computational sciences to refine disease diagnosis, prognosis, and treatment selection. Unlike conventional medicine, which applies standardized interventions across broad patient populations, precision medicine accounts for inter-individual variability in drug response and disease susceptibility, thereby enhancing therapeutic efficacy while minimizing adverse effects [1].

The significance of precision medicine is particularly evident in oncology and metabolic disorders, where genetic heterogeneity and molecular complexity contribute to highly variable treatment responses. In oncology, tumor heterogeneity necessitates the identification of genetic markers that predict therapeutic outcomes, facilitating pharmacogenomics-driven stratification of cancer patients for personalized chemotherapy, immunotherapy, and targeted treatments. This individualized approach improves treatment efficacy, mitigates toxicity, and enhances patient survival. Similarly, in metabolic disorders such as diabetes and obesity, genetic predispositions influence drug metabolism, insulin sensitivity, and lipid regulation, necessitating precision-based interventions to optimize therapeutic responses and prevent disease progression [2].

Advancements in high-throughput sequencing, artificial intelligence (AI), and gene-editing technologies are driving unprecedented progress in precision medicine. The convergence of pharmacogenomics, AI, and CRISPR-based genome editing has transformed drug discovery, therapeutic gene modification, and patient-specific treatment strategies. Pharmacogenomics enables the identification of genetic variants that regulate drug absorption, metabolism, and efficacy, thereby refining pharmacokinetics and pharmacodynamics to optimize treatment selection [3]. AI, particularly machine learning (ML) and deep learning (DL) enhances the analysis of high-dimensional genomic datasets, predicts patient-specific drug responses, and refines biomarker discovery while accelerating drug repurposing strategies. CRISPR-Cas genome editing has revolutionized therapeutic interventions by enabling precise modification of genomic sequences, with significant implications for correcting oncogenic mutations, improving immunotherapy, and modulating metabolic pathways implicated in insulin resistance and lipid homeostasis [4].

**Figure 1:**
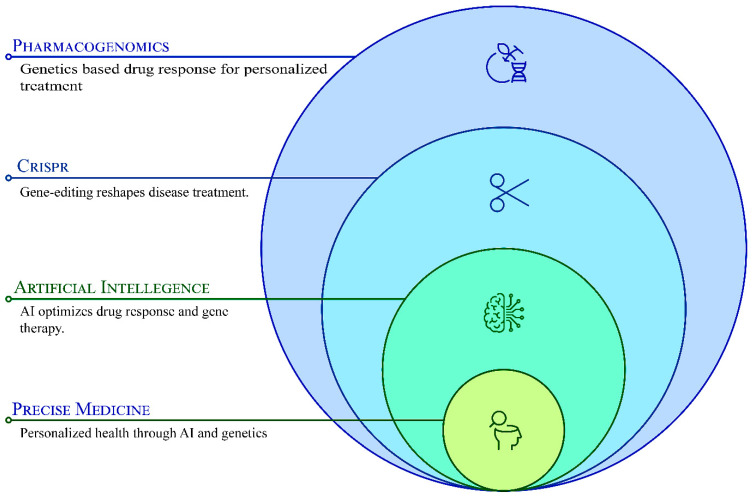
The synergistic integration of pharmacogenomics, CRISPR genome editing, and artificial intelligence (AI) in driving advancements in precision medicine.

Integrating AI with pharmacogenomics and CRISPR has refined precision medicine by improving drug–gene interaction predictions, optimizing gene-editing specificity, and advancing predictive modeling for therapeutic responses (Figure 1). AI algorithms enhance CRISPR guide RNA (gRNA) design, reducing off-target effects and improving editing precision, while pharmacogenomic insights inform the selection of CRISPR-based interventions for personalized disease management. This interdisciplinary approach is paving the way for AI-augmented gene therapy and pharmacogenomics-driven drug development, ultimately transforming the landscape of individualized treatment [5]. Despite these advancements, significant challenges remain in translating pharmacogenomic insights and gene-editing technologies into clinical applications.

The complexity of genetic variations, biases in AI-driven predictive models, ethical concerns surrounding genome editing, and regulatory constraints hinder the widespread adoption of AI-CRISPR-based therapeutics [6].

This review comprehensively analyzes the interplay between AI, pharmacogenomics, and CRISPR in precision medicine, particularly in oncology and metabolic diseases. It examines the role of pharmacogenomic insights in guiding precision therapy, the impact of AI in predicting drug responses and biomarker discovery, and the potential of CRISPR-based interventions in correcting pathogenic mutations and modulating metabolic pathways. Furthermore, this review discusses emerging trends in AI-assisted drug discovery and genome editing, highlights algorithmic biases, ethical dilemmas, and data privacy concerns, and evaluates regulatory and translational barriers in CRISPR-based therapeutics.

## 2. Pharmacogenomics: The Foundation of Precision Medicine

### 2.1. Genetic Variability and Its Role in Drug Response

Pharmacogenomics plays a pivotal role in precision medicine by elucidating how genetic variability influences drug metabolism, efficacy, and toxicity. Inter-individual differences in drug response can be attributed to genetic variations that affect pharmacokinetics (drug absorption, distribution, metabolism, and excretion) and pharmacodynamics (interaction with target receptors, enzymes, or transporters). These genetic differences, primarily in the form of single nucleotide polymorphisms (SNPs), copy number variations (CNVs), and structural rearrangements, significantly impact drug metabolism by altering the function of critical enzymes and transporters. Studying these genetic factors enables clinicians to tailor drug therapies to an individual’s genetic profile, thereby enhancing therapeutic outcomes and reducing adverse drug reactions (ADRs) [7].

Drug metabolism is primarily governed by a complex interplay of genetic factors within the cytochrome P450 (CYP) enzyme family, phase II conjugation enzymes, and membrane-bound transporters. These enzymes and transporters regulate the biotransformation and clearance of drugs, ultimately influencing their bioavailability and therapeutic efficacy. The genetic architecture of drug metabolism is shaped by an individual’s haplotype structure, comprising specific genetic variants inherited within a population [7].

Genetic polymorphisms in drug-metabolizing enzymes can lead to functional consequences such as ultrarapid metabolism (UM), extensive metabolism (EM), intermediate metabolism (IM), or poor metabolism (PM). For instance, polymorphisms in CYP2D6 determine the rate at which drugs like tamoxifen, codeine, and tricyclic antidepressants are metabolized. Individuals carrying multiple copies of functional CYP2D6 alleles exhibit ultrarapid metabolism, leading to insufficient therapeutic effects due to rapid drug clearance, whereas poor metabolizers accumulate the drug, predisposing them to toxicity. Similarly, variations in CYP2C9 and VKORC1 significantly affect warfarin metabolism, necessitating genotype-guided dosing to minimize bleeding risks [7,8].

The pharmacogenomics of drug transporters also plays a crucial role in drug response variability. Variants in ABCB1 (P-glycoprotein) can influence drug efflux, leading to altered plasma drug concentrations. For instance, mutations in SLCO1B1, which encodes the organic anion-transporting polypeptide 1B1 (OATP1B1), have been linked to increased statin-induced myopathy, demonstrating how transporter polymorphisms can impact drug disposition and toxicity [9].

### 2.2. Pharmacogenetic Markers: Genetic Variants Influencing Drug Metabolism

Pharmacogenetic markers are crucial in determining an individual’s ability to metabolize drugs, affecting their efficacy and safety. These markers are primarily genetic polymorphisms in drug-metabolizing enzymes, which can significantly alter pharmacokinetics by influencing drug absorption, metabolism, and clearance. The metabolism of drugs is categorized into Phase I and Phase II metabolic reactions, with Phase I primarily involving oxidation, reduction, and hydrolysis. At the same time, Phase II is responsible for conjugation reactions such as methylation, acetylation, and glucuronidation. Genetic polymorphisms within the genes encoding these metabolic enzymes can result in varied enzymatic activity, ranging from ultrarapid metabolism (UM) to poor metabolism (PM), impacting drug response in individuals [10].

#### 2.2.1. Functional Classification of Pharmacogenes

##### Phase I Metabolism Genes (Oxidation, Reduction, Hydrolysis)

**Figure 2:**
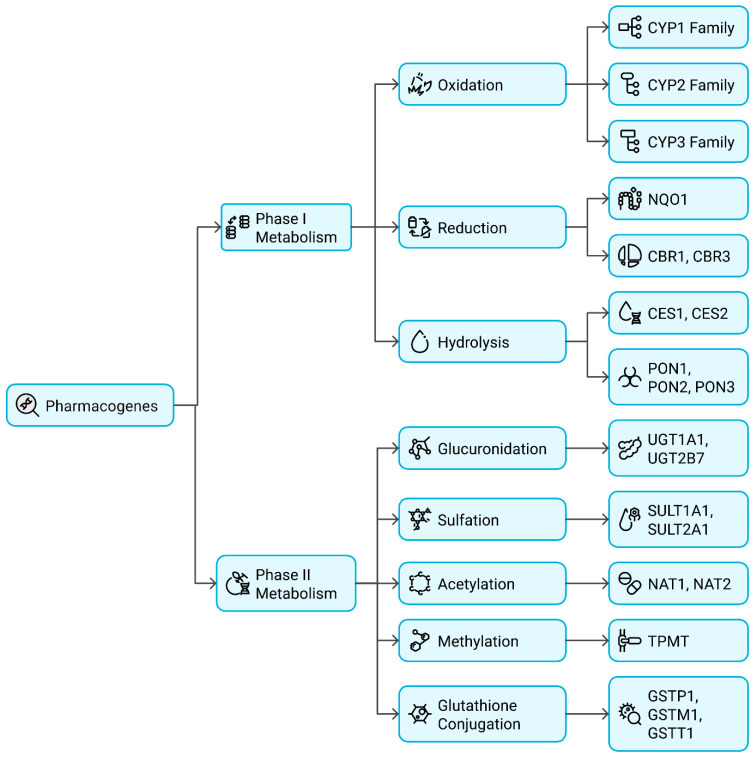
The functional classification of pharmacogenes based on metabolic pathways, distinguishing Phase I enzymes (Oxidation, Reduction, Hydrolysis), from Phase II enzymes (Conjugation Reactions).

Mostly mediating Phase I drug metabolism, the Cytochrome P450 (CYP) enzyme family is responsible for the oxidative biotransformation of roughly 75–80% of clinically used drugs (Figure 2). By altering CYP enzyme polymorphisms, one can significantly vary drug clearance rates and either produce therapeutic failure or toxicity. Anticoagulants and antiplatelet drugs are metabolized by CYP2C9 and CYP2C19, respectively. CYP2C9 is especially important in warfarin metabolism, where polymorphic variants like CYP2C92 reduce enzyme activity, so influencing warfarin clearance. Patients having these alleles require less warfarin to avoid bleeding issues. Similar effects of CYP2C19 polymorphisms influence the bioactivation of clopidogrel, a prodrug indicated to prevent platelet aggregation. In those on antiplatelet therapy, loss-of-function alleles (CYP2C192 and 3) cause inadequate drug activation, hence increasing their risk of cardiovascular events [11].

Another crucial enzyme known as CYP2D6 metabolizes several psychotropic drugs, including codeine (an opioid analgesic) and tamoxifen (used in breast cancer treatment). Wide variation in the CYP2D6 gene covers gene duplications (ultrarapid metabolizers) to non-functional alleles (poor metabolizers). CYP2D6-poor metabolizers of tamoxifen are unable to efficiently convert tamoxifen into its active metabolite, endoxifen, so they reduce therapeutic efficacy. Conversely, ultrarapid metabolizers run too great a danger of side effects because of their too high medication conversion. Quickly converting opioids, including codeine, to morphine, CYP2D6 ultrarapid metabolizers raise their toxicity and risk of respiratory depression [12].

Immunosuppressant and statin breakdown is much aided by the CYP3A4 and CYP3A5 enzymes. Tacrolimus, a key immunosuppressant used in organ transplantation, is metabolized by CYP3A5; dosage needs to follow from this. While those expressing CYP3A51 need more tacrolimus, those carrying CYP3A53 have reduced metabolism rates, which causes drug accumulation and increased nephrotoxicity risk. Moreover, affecting the metabolism of statins, particularly atorvastatin and simvastatin, CYP3A4 polymorphisms so modify the efficacy of lipid-lowering treatment and myopathy risk [13].

##### Phase II Metabolism Genes (Conjugation Reactions)

Phase II metabolism primarily involves conjugation reactions that enhance drug solubility, facilitating excretion (Figure 2). These reactions include glucuronidation, methylation, and acetylation, mediated by specific enzymes, including UDP-glucuronosyltransferases (UGTs), Thiopurine Methyltransferase (TPMT), and N-Acetyltransferase (NAT). UDP-glucuronosyltransferases (UGTs) catalyze the glucuronidation of several drugs, aiding in their elimination. One of the most clinically significant UGT enzymes is UGT1A1, which metabolizes irinotecan, a chemotherapy agent used in colorectal cancer treatment. Individuals carrying the UGT1A128 variant allele have reduced enzyme activity, leading to impaired drug clearance and an increased risk of severe neutropenia (life-threatening bone marrow suppression). As a result, dose modifications are recommended for patients with UGT1A1 polymorphisms undergoing irinotecan therapy [11].

Thiopurine Methyltransferase (TPMT) is crucial in metabolizing thiopurine drugs, such as azathioprine and mercaptopurine, used in cancer treatment and autoimmune disorders. TPMT polymorphisms influence the enzymatic breakdown of thiopurines, where individuals carrying TPMT2, 3A, and 3C variants exhibit significantly reduced enzyme activity. This results in the accumulation of toxic metabolites, leading to myelosuppression and bone marrow toxicity. Pharmacogenomic testing for TPMT variants before thiopurine therapy can help personalize dosing strategies to prevent severe adverse effects [12].

N-acetyltransferase 2 (NAT2) is crucial in metabolizing several drugs, including isoniazid, used in tuberculosis (TB) treatment. NAT2 polymorphisms classify individuals as slow, intermediate, or rapid acetylators, significantly impacting isoniazid clearance. Slow acetylators (carriers of NAT2 slow-activity alleles) are at an increased risk of drug-induced hepatotoxicity and peripheral neuropathy. In contrast, rapid acetylators clear the drug more efficiently but may experience treatment failure due to subtherapeutic drug levels [13].

### 2.3. Mechanistic Insights into Pharmacogenomic Variations

**Figure 3:**
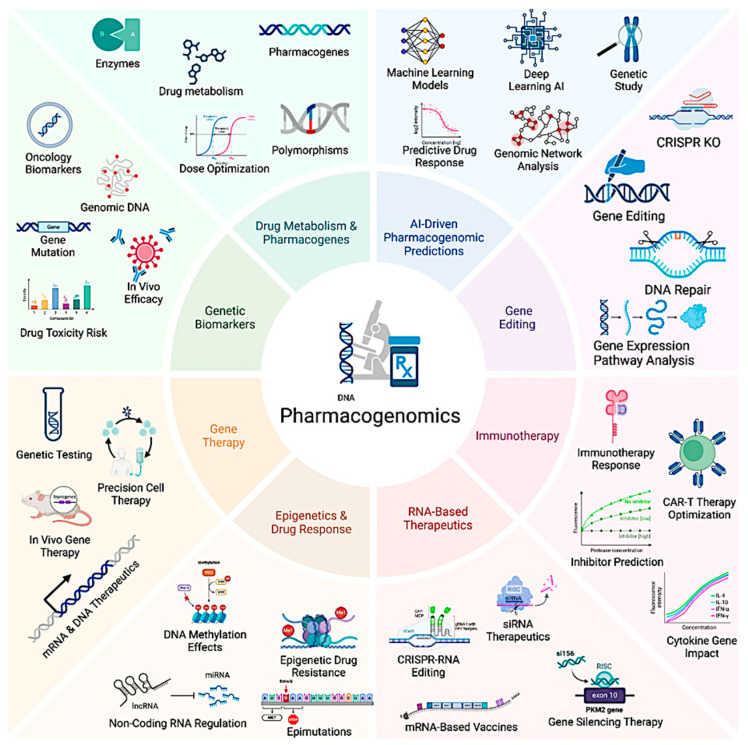
The schematic figure presents a holistic overview of pharmacogenomics. It depicts the integration of emerging technologies and therapeutic strategies in precision medicine. The key exploration of how genetic variability influences drug metabolism, efficacy, and toxicity. Key domains include pharmacogenes, dose optimization, AI-driven drug response prediction, CRISPR-based genome editing, and immunotherapy refinement. The figure further highlights RNA-based therapeutics (siRNA, mRNA vaccines), epigenetic modulation, and gene therapy approaches for personalized treatment or therapy. Genetic biomarker identification, including gene mutations and toxicity risk assessment, enables tailored interventions. (Created in BioRender 201).

Pharmacogenomic variations are pivotal in shaping inter-individual differences in drug metabolism and response. These variations can occur at multiple genomic levels, influencing gene expression, enzyme activity, and drug disposition (Figure 3). The three major mechanisms of pharmacogenomic variability are single nucleotide polymorphisms (SNPs), copy number variations (CNVs), and epigenetic modifications. SNPs represent the most common type of genetic variation, altering amino acid sequences and regulatory elements that control gene expression. CNVs involve large-scale genomic alterations, such as gene duplications or deletions, leading to significant variations in enzyme activity levels. Additionally, epigenetic modifications, including DNA methylation, histone modifications, and microRNA (miRNA)-mediated regulation, dynamically alter gene expression without modifying the DNA sequence itself. These mechanisms collectively impact drug metabolism pathways, influencing therapeutic efficacy and the risk of adverse drug reactions (ADRs) [14].

#### 2.3.1. Functional Consequences of Single Nucleotide Polymorphisms (SNPs)

Comprising roughly once per 300–1000 base pairs in the human genome, SNPs are the most common kind of genetic variation. Depending on their position and effect on mRNA splicing, protein structure, or gene transcription, these single-base pair changes can have different functional effects. Drug pharmacokinetics and pharmacodynamics were substantially influenced by SNPs in pharmacogenes, especially those coding for transporters and drug-metabolizing enzymes [15].

##### Pharmacogenes: Synonymous vs. Non-Synonymous SNPs

Silent mutations, sometimes referred to as synonymous SNPs, arise from the genetic code’s redundancy causing a nucleotide replacement not to change the encoded amino acid. Although historically thought to be functionally neutral, new data indicates that synonymous SNPs can change mRNA stability, translation efficiency, and protein folding, therefore influencing drug metabolism. One such a synonymous mutation linked with changed P-glycoprotein expression, therefore affecting the bioavailability of chemotherapeutic drugs and immunosuppressants. ABCB1 C3435T is one example. Conversely, non-synonymous SNPs either cause premature stop codons (nonsense mutations) or amino acid substitutions (missense mutations), therefore changing the enzyme activity. For example, CYP2C92 (Arg144Cys) and CYP2C93 (Ile359Leu) greatly lower CYP2C9 enzyme activity, therefore affecting warfarin metabolism and raising bleeding risk. Analogously, the CYP2D64 variant splice-site mutation results in a non-functional enzyme influencing the metabolism of opioids and antidepressants [16].

##### Drug Response Splice Site and Frameshift Mutations

At exon-intron junctions, splice site mutations alter the normal splicing of pre-mRNA and may either include or exclude important coding sequences. Such mutations can produce shortened or defective proteins, therefore profoundly affecting drug metabolism. One such erroneous splicing site introduced by the CYP2C19*2 allele generates a non-functional enzyme unable of bioactivating clopidogrel. Poor metabolizers’ increased risk of cardiovascular events and clopidogrel resistance follow from this [17]. Insertions or deletions (indels) of nucleotides produce frameshift mutations that cause a change in the reading frame and early termination of translation. Usually generating non-functional proteins, these mutations can have significant pharmacogenomic effects. One well-defined example is the DPYD*2A mutation, which causes dihydropyrimidine dehydrogenase (DPD) impairment, therefore lowering the metabolism of fluoropyrimidine-based treatment (e.g., 5-fluorouracil). Patients with this variation are very prone to severe toxicity, hence genotype-guided dosage changes are quite important [18].

#### 2.3.2. Copy Number Variations (CNVs) in Drug Metabolism

CNVs refer to large-scale genomic alterations, including gene duplications, deletions, and rearrangements, leading to significant inter-individual variability in drug metabolism. CNVs affecting pharmacogenes can increase or decrease enzyme expression levels, profoundly influencing drug efficacy and safety. A key example of CNVs in pharmacogenomics is the CYP2D6 gene, which exhibits extreme inter-individual variation due to gene duplications or deletions. CYP2D6 ultra-rapid metabolizers (UMs) carry multiple copies of the functional gene (CYP2D62xN), leading to excessive drug metabolism. This is particularly relevant in opioid therapy, where ultra-rapid metabolizers convert codeine into morphine at an accelerated rate, increasing the risk of respiratory depression and opioid toxicity. Conversely, CYP2D6-poor metabolizers (PMs), which carry non-functional alleles (CYP2D63, CYP2D64, CYP2D65), experience reduced drug metabolism, resulting in inadequate analgesic effects or excessive accumulation of antidepressants, increasing side effects such as QT prolongation and serotonin toxicity [19].

Beyond CYP2D6, CNVs in UGT2B17, encoding a UDP-glucuronosyltransferase enzyme involved in steroid metabolism, have been linked to variable drug glucuronidation rates. The deletion of UGT2B17 has been associated with altered testosterone metabolism and variable clearance of nonsteroidal anti-inflammatory drugs (NSAIDs), affecting their efficacy and duration of action [20].

#### 2.3.3. Epigenetic Modifications and Drug Reactivity

Epigenetic mechanisms include DNA methylation, histone modifications, and microRNA (miRNA)-mediated regulation are very essential for modulating gene expression in response to environmental events including drug exposure. Unlike genetic changes, epigenetic ones are dynamically regulated and reversible, therefore adding still another degree of complexity to pharmacogenomic variability [17].

##### MiRNA Control and DNA Methylation of Drug-Metabolizing Enzymes

Usually inhibiting gene expression, DNA methylation adds a methyl group (-CH_3_) to cytosine residues in CpG islands. Hypermethylation of promoter areas of drug-metabolic enzyme genes can reduce transcription, therefore reducing enzyme activity. One such a CYP3A4 gene whereby promoter methylation downregulates enzyme synthesis, hence influencing medication clearance and immunosuppressant response [18]. MiRNAs, small non-coding RNAs that regulate gene expression post-transcriptionally, are emerging as major controllers of pharmacogenomic variation. Targeting CYP3A4 and CYP2C19, certain miRNAs such as miR-27b and miR-148a alter enzyme levels, therefore affecting drug metabolism. MiR-200c downregulation enhances ABCB1 (P-glycoprotein) expression in cancer pharmacogenomics, therefore encouraging drug efflux from tumor cells and hence treatment resistance [21].

##### Gene Expression and Histone Modifications in Pharmacogenomics

Histone changes affect chromatin shape and transcriptional activity including acetylation, methylation, and phosphorylation. Whereas histone acetylation, mediated by histone acetyltransferases (HATs), loosens chromatin and increases gene transcription, histone deacetylation (HDAC) condenses chromatin and hence reduces gene expression. Currently under research as HDAC inhibitors include vorinostat [22] epigenetic drugs to modulate pharmacogenes and prevent drug resistance in cancer treatment. One well-known example of histone modification influencing drug metabolism is the epigenetic modulation of CYP1A1, an enzyme involved in carcinogen metabolism. Under the CYP1A1 promoter, polycyclic aromatic hydrocarbons (PAHs) histone acetylate smokers, hence increasing enzyme expression and pro-carcinogen bioactivation. Understanding such epigenetic mechanisms opens the path for targeted treatments in individualized medicine [23].

## 3. CRISPR Genome Editing: A Revolutionary Tool in Personalized Medicine

### 3.1. CRISPR and Its Role in Precision Medicine

CRISPR (Clustered Regularly Interspaced Short Palindromic Repeats) gene-editing technology has revolutionized genetic engineering by offering an efficient, precise, and scalable method for modifying genomic sequences. Discovered initially as part of the bacterial immune system, CRISPR-Cas systems have been adapted into powerful tools for genome manipulation in eukaryotic cells. This breakthrough technology has significantly enhanced our ability to study gene function, correct disease-associated mutations, and develop personalized therapeutic strategies. Unlike earlier genome-editing methods, such as zinc finger nucleases (ZFNs) and transcription activator-like effector nucleases (TALENs), CRISPR-Cas systems provide a more versatile and user-friendly approach to targeted gene modification [24].

The CRISPR-Cas9 system, first described in 2012, is the most widely utilized gene-editing tool due to its ability to introduce double-stranded breaks (DSBs) at specific genomic loci guided by a programmable single-guide RNA (sgRNA). Upon DNA cleavage, the cellular repair machinery employs either non-homologous end joining (NHEJ), which often introduces insertions or deletions (indels) leading to gene knockout, or homology-directed repair (HDR), which facilitates precise gene correction when a homologous repair template is available. These capabilities make CRISPR-Cas9 highly valuable for applications in gene therapy, functional genomics, and drug development [15].

In addition to CRISPR-Cas9, other CRISPR-associated nucleases have been developed to expand the scope of genome editing, each with unique properties that enhance precision medicine applications. CRISPR-Cas12 and CRISPR-Cas13 are two alternative systems that offer distinct advantages over Cas9, particularly in terms of specificity and RNA-targeting capabilities (Table 1) [16].

### 3.2. Mechanistic Insights into CRISPR Genome Editing

The CRISPR-Cas9 system has emerged as a revolutionary genome-editing tool due to its ability to introduce precise genetic modifications at specific genomic loci. Unlike earlier genome-editing approaches that relied on complex protein engineering, CRISPR functions through a programmable guide RNA (gRNA) that directs the Cas9 endonuclease to a complementary target DNA sequence. The ability to target virtually any gene by modifying the gRNA sequence has made CRISPR an indispensable tool in functional genomics, precision medicine, and gene therapy [17].

**Figure 4:**
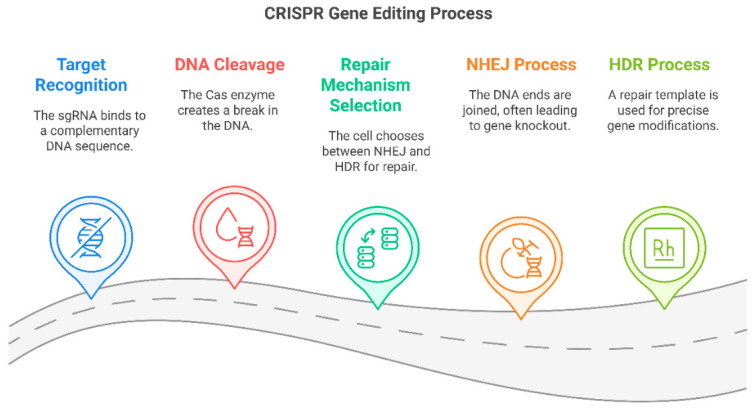
The CRISPR genome editing process and its mechanism of DNA modification.

At the molecular level, CRISPR-mediated genome editing primarily occurs through two distinct DNA repair pathways following the introduction of a double-stranded break (DSB) by Cas9: Non-Homologous End Joining (NHEJ) and Homology-Directed Repair (HDR) (Figure 4). Each pathway has distinct functional consequences and applications, determining whether CRISPR will be used for gene disruption, correction, or insertion of new genetic sequences. In addition to classical CRISPR-Cas9 editing, novel refinements, such as base editing and prime editing, have been developed to enhance the specificity and efficiency of genetic modifications. These approaches circumvent the need for creating DSBs, thereby reducing off-target effects and unintended mutations [18].

#### 3.2.1. Molecular Mechanism of CRISPR-Cas9

The CRISPR-Cas9 gene-editing system is derived from the adaptive immune system of bacteria and archaea, which functions as a defense mechanism against viral infections. In its natural setting, bacteria incorporate fragments of viral DNA (spacers) into their CRISPR loci, allowing them to recognize and target invading viruses upon subsequent exposure. The Cas9 endonuclease, guided by a complementary gRNA, binds to the target DNA and induces a double-stranded break (DSB) at a precise genomic location. This sequence-specific cleavage is the foundation for genome editing in mammalian cells [21].

**Figure 5:**
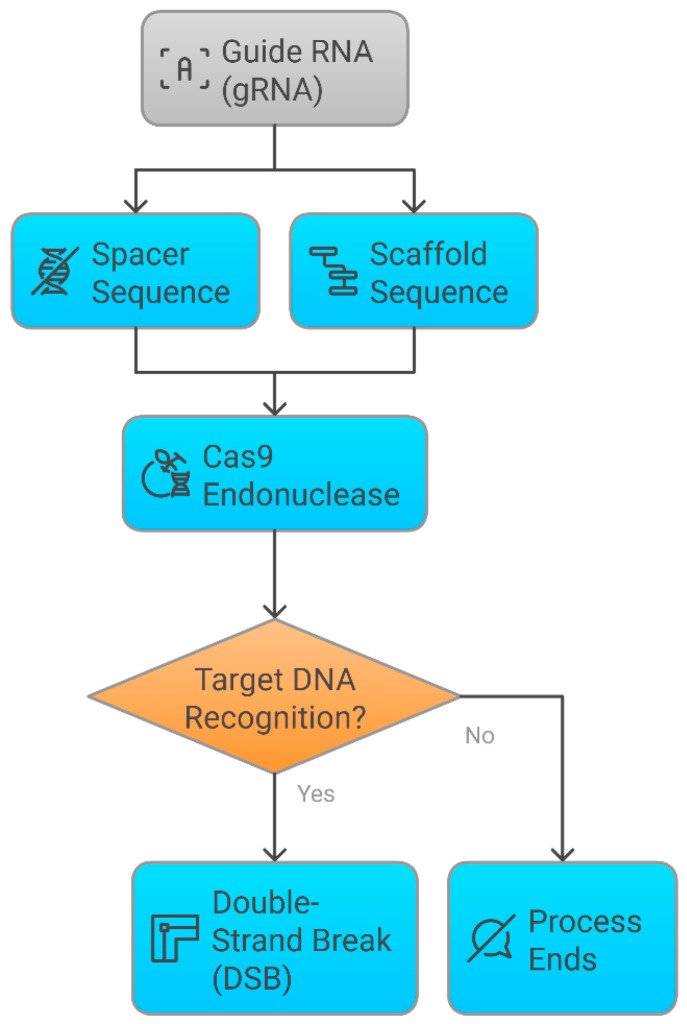
The Figure illustrates the CRISPR-Cas9 genome editing mechanism. A guide RNA (gRNA), comprising a spacer and scaffold sequence, directs the Cas9 endonuclease to specific DNA target. Where upon successful DNA recognition, Cas9 induces a site-specific double-strand break (DSB). This mechanism underlies precise genome editing applications in biomedical research and therapy.

The CRISPR-Cas9 system consists of two primary components: Guide RNA (gRNA), A synthetic, programmable RNA sequence that directs Cas9 to a specific protospacer-adjacent motif (PAM) containing DNA sequence. The gRNA consists of two regions: The spacer sequence (~20 nucleotides) complementary to the target DNA. The scaffold sequence facilitates Cas9 binding and stabilization. Second, Cas9 Endonuclease: A RNA-guided DNA-cleaving enzyme that recognizes and binds to the target DNA site immediately upstream of a PAM sequence (typically 5′-NGG-3′ for *Streptococcus pyogenes* Cas9). Upon target recognition, Cas9 induces a double-strand break (DSB) at the target locus, triggering DNA repair pathways (Figure 5) [22].

#### 3.2.2. Limitations of Subcellular Delivery in CRISPR Genome Editing

Despite the transformative promise of CRISPR-based genome engineering, its clinical translation is significantly hindered by challenges associated with the efficient and targeted subcellular delivery of gene-editing components. Successful genome editing requires the precise transport of the CRISPR-Cas9 system comprising the Cas9 protein or its messenger RNA (mRNA) and the single-guide RNA (sgRNA) across multiple biological barriers, including the plasma membrane, cytosol, and nuclear envelope. Each of these steps presents unique technical and physiological obstacles that impact editing efficiency, specificity, and therapeutic safety [23].

CRISPR delivery systems are broadly categorized into viral and non-viral vectors, each with intrinsic limitations. Adeno-associated viruses (AAVs) have gained popularity due to their low immunogenicity and their ability to transduce both dividing and non-dividing cells in vivo. However, the limited packaging capacity of AAV (~4.7 kb) constrains their utility for delivering larger CRISPR systems such as Streptococcus pyogenes Cas9 (SpCas9) along with regulatory elements and sgRNA. Lentiviral and retroviral vectors offer larger cargo capacities and stable genomic integration, but their use raises concerns regarding insertional mutagenesis, oncogenic potential, and prolonged Cas9 expression, which can inadvertently increase off-target editing [25].

Non-viral vectors, including lipid nanoparticles (LNPs), have emerged as promising alternatives due to their ability to enable transient expression and reduce the risk of genotoxicity. Still, these systems sometimes have inadequate endosomal escape, which causes the CRISPR cargo within endolysosomal compartments to be enzymatically broken down. Though cytotoxicity, immunological activation, and particle aggregation still restrict their translational potential, additional non-viral platforms including cell-penetrating peptides, polymeric carriers, and gold nanoparticles are presently under investigation. Although ex vivo genome editing still mostly uses electroporation, especially in CAR-T cell therapies, its invasive character and restricted applicability to in vivo systems hinder more general therapeutic use [26].

Beyond cell entrance, intracellular trafficking creates more obstacles. Once in the cytoplasm, CRISPR elements have to effectively pass the nuclear membrane to reach genomic DNA. In non-dividing cells, where the nuclear envelope stays whole, that stage is very challenging. Although Cas9 is usually built with nuclear localization signals (NLS), the significance of ribonucleoprotein (RNP) complexes into the nucleus stays ineffective. Furthermore, greatly reducing the bioavailability of CRISPR machinery is an intracellular breakdown and trapping in cytosolic vesicles, particularly in physiologically complicated or hard-to-transfect cell types such as neurons and immunological cells [26].

Moreover, achieving tissue-specific delivery remains an ongoing challenge. Systemic administration of CRISPR components often results in heterogeneous transfection efficiencies across tissues, largely influenced by receptor expression, membrane properties, and local microenvironments. For instance, hepatocytes are efficiently targeted by LNPs due to their interaction with apolipoprotein E (ApoE), facilitating uptake in the liver. In contrast, effective delivery to tissues such as the brain, pancreas, or skeletal muscles remains elusive. Physiological barriers such as the extracellular matrix, immune surveillance, and the blood–brain barrier (BBB) further limit access to immune-privileged and fibrotic tissues [27].

Finally, immunogenicity and cytotoxicity pose critical safety concerns. Cas9 orthologs, particularly SpCas9, are derived from bacterial sources and may trigger innate and adaptive immune responses upon in vivo administration. Pre-existing immunity or the development of immune responses following repeated dosing can reduce editing efficacy and provoke inflammatory complications. In parallel, delivery vehicles including cationic lipids and synthetic polymers can induce dose-dependent cytotoxicity and inflammatory responses, thereby limiting their suitability for clinical applications requiring repeated or long-term intervention [28].

#### 3.2.3. Base Editing and Prime Editing: Next-Generation CRISPR Approaches

Although genome engineering depends on conventional CRISPR-Cas9 editing, the creation of DSBs generates major issues, including off-target mutagenesis, accidental massive deletions, and chromosomal rearrangement. Novel approaches, such as base editing and prime editing, have been developed to solve these issues and enable accurate single-nucleotide changes without generating DSB [29]. One single-nucleotide alteration technique known as “base editing” involves the direct changing of one DNA base to another without producing a DSB or using a donor template. This method mostly helps fix point mutations linked to genetic disorders. Cytosine Base Editors (CBEs), for instance, are programmed to change cytosine (C) to thymine (T). These comprise a catalytically dead Cas9 (dCas9) coupled to a cytidine deaminase enzyme to allow direct deamination of cytosine to uracil, hence producing a C to T substitution during DNA replication. In β-thalassemia, Duchenne muscular dystrophy (DMD), and sickle cell anemia, CBEs have effectively fixed harmful mutations. Although they mediate adenine (A) to guanine (G) conversions via an engineered adenine deaminase enzyme, the Adenine Base Editors (ABEs) behave similarly to CBEs. Correcting G to A transition mutations, the most usually occurring harmful mutation in genetic illnesses, calls especially for this capacity. Preclinical models of metabolic diseases, muscular dystrophy, and hereditary deafness have been using ABEs [30].

### 3.3. CRISPR in Cancer Therapy and Precision Oncology

**Figure 6:**
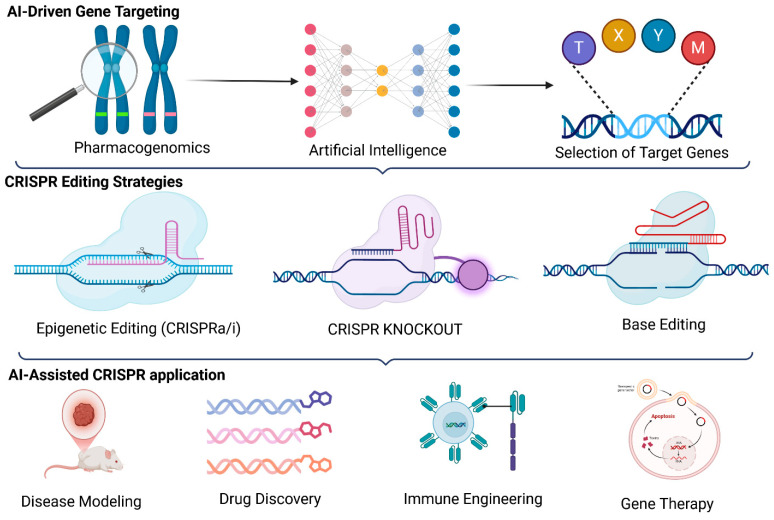
AI-driven CRISPR genome editing approach for precision medicine, showcasing how AI enhances gRNA design, predicts off-target effects, and integrates pharmacogenomic and multi-omics data to optimize genome editing, improve therapeutic outcomes, and advance applications in disease modeling, drug discovery, immunotherapy, and regenerative medicine. (Created in BioRender 201).

Cancer is a highly heterogeneous disease driven by genetic and epigenetic alterations that influence tumor growth, metastasis, and treatment resistance. Advances in genome sequencing have revealed oncogenic mutations and dysregulated signaling pathways, enabling the development of targeted cancer therapies. However, many cancers exhibit intratumoral genetic heterogeneity and develop drug resistance over time, limiting the efficacy of existing treatments. The emergence of CRISPR-Cas9 genome editing has revolutionized cancer research and therapy by allowing the precise modification of oncogenic mutations, tumor suppressor genes, and immune checkpoint regulators. CRISPR has become essential in functional oncology, enabling researchers to study cancer-driving mutations, screen for therapeutic targets, and enhance immunotherapy strategies (Figure 6) [31].

#### 3.3.1. Targeting Oncogenic Mutations for Personalized Cancer Treatment

Genetic alterations in oncogenes (KRAS, EGFR, MYC) and tumor suppressor genes (TP53, PTEN, BRCA1/2) drive the majority of tumorigenesis. These changes assist in clarifying chemotherapeutic resistance, death avoidance, and unchecked cell proliferation. Providing fresh routes for tailored cancer treatment, CRISPR has provided a precise technique to edit or fix oncogenic mutations [32].

One of the most thoroughly studied oncogenic mutations is found in KRAS, a gene coding a small GTPase that is important in cell signaling. Commonly displaying KRAS mutations, especially KRAS G12D and G12V which promote constitutive activation of the RAS-RAF-MEK-ERK signaling cascade are pancreatic, colorectal, and lung cancers. The deletion or base editing of mutant KRAS driven by CRISPR-Cas9 was investigated as a therapeutic method to inhibit tumor growth and increase chemotherapy sensitivity. Focusing exclusively on mutant KRAS will help researchers to minimize off-target toxicity and widen the treatment window by preserving the wild-type allele [33].

Another key use for CRISPR in cancer is TP53, a fundamental tumor suppressor commonly known as the “guardian of the genome”. TP53 mutations induce dysfunction and defective DNA damage response systems in over 50% of malignant cancers. Using CRISPR, reactive wild-type TP53 expression or repaired loss-of-function TP53 mutations have been reactivated, hence restoring normal death processes in cancer cells. CRISPR-Cas9 screening studies have also shown synthetic lethal interactions involving TP53, therefore highlighting fresh therapeutic vulnerabilities in TP53-mutant tumors [34].

EGFR (Epidermal Growth Factor Receptor) mutations cause oncogenic signaling in non-small cell lung cancer (NSCLC) and glioblastoma, hence increasing tumor growth and resistance against EGFR tyrosine kinase inhibitors (TKIs). Specifically removing defective EGFR alleles, CRISPR-based genome editing has turned off aberrant growth signals. Moreover, high-throughput genomic screens enabled by CRISPR have helped to identify resistance mechanisms in EGFR-mutant tumors, therefore enabling the development of next-generation targeted treatments [35].

Beyond direct genome editing, CRISPR technology is being applied with immune checkpoint blockade therapy to increase T-cell-based cancer immunotherapy efficacy. By perturbing immune inhibitory genes such as PD-1 (Programmed Death-1) and CTLA-4 (Cytotoxic T-Lymphocyte Antigen-4) in T cells, CRISPR can stimulate anti-tumor immune responses, hence enhancing the therapeutic outcomes of immune checkpoint inhibitors (ICIs) in resistant tumors [36].

#### 3.3.2. CRISPR-Modified CAR-T Cells in Immunotherapy

Chimeric antigen receptor T-cell (CAR-T) therapy has become a transforming weapon in cancer immunotherapy especially for hematologic malignancies like acute lymphoblastic leukemia (ALL), diffuse large B-cell lymphoma (DLBCL), and multiple myeloma. Genetically modified T cells with an artificial tumor antigen-specific receptor let them especially recognize and kill cancer cells selectively. Among the various difficulties often encountered in CAR-T cell treatment are immune evasion, T-cell depletion, and antigen escape. Fresh approaches to improve CAR-T cell performance, persistence, and specificity are provided by CRISPR genome editing [37].

Eliminating immunological checkpoint controllers as PD-1 and CTLA-4 is among the most interesting uses for CRISPR in CAR-T treatment. Usually speaking, malignancies resist immunological attack by upregulating PD-L1 (Programmed Death-Ligand 1), which binds to PD-1 on T cells, therefore reducing their activity. Researchers have produced PD-1 knockout CAR-T cells with increased tumor-killing capacity and extended persistence in the tumor microenvironment by way of CRISPR disruption of the PD-1 gene in CAR-T cells. Overcoming immune checkpoint medicines, preclinical results show that CRISPR-PD-1 deletion CAR-T cells boost responses in PD-L1-expressing malignancies including lung and liver cancer [38]. Moreover, CAR-T cell treatments benefit in production and safety profile by means of CRISPR. Under conventional CAR-T treatment, a time-consuming and expensive process, patient-derived autologous T cells are genetically transformed to express CARs. By deleting endogenous T-cell receptor (TCR) genes, CRISPR-Cas9 produces allogeneic (off-the-shelf) universal CAR-T cells, hence preventing graft-versus-host disease (GVHD), and so enabling donor-derived CAR-T therapy. For a larger patient population, this strategy can lower production costs, boost access, and improve therapy scalability [39].

Beyond hematologic cancers, solid tumors where immunosuppressive tumor microenvironments and inadequate T-cell penetration have limited efficacy are being changed for CAR-T treatment augmented by CRISPR. Improved CAR-T cell trafficking, survival, and tolerance to immunosuppressive signals follow from CRISPR changes. For instance, CRISPR has been used to alter chemokine receptors (CXCR3, CCR5) on CAR-T cells so enhancing their homing ability to solid tumor locations. Furthermore, overcoming immunological inhibition, CAR-T cell design using CRISPR releases IL-12 or IL-15 cytokines, thus increasing their proliferation and persistence inside the tumor microenvironment [40].

By using dual-targeting CAR-T cell technologies in which T cells are altered to concurrently identify several tumor antigens, CRISPR has been made available, therefore lowering the probability of antigen escape. For models of breast and ovarian cancer, for example, dual CAR-T cells produced by CRISPR targeting HER2 and MUC1 have shown increased anti-tumor efficacy. These developments show great promise to extend CAR-T therapy from hematologic malignancies to solid tumors, therefore satisfying a significant unmet need in cancer immunotherapy [41].

### 3.4. CRISPR for Neurological and Metabolic Disorders in Precision Medicine

Precision medicine’s transforming approach for neurological and metabolic diseases is now CRISPR-based genome editing. Many times, arising from particular genetic abnormalities leading to neuronal malfunction and neurodegeneration are neurological illnesses, including Alzheimer’s disease (AD), Huntington’s disease (HD), and Parkinson’s disease (PD). Likewise, substantial genetic determinants of metabolic diseases, including diabetes, obesity, and hypercholesterolemia, influence insulin signaling, lipid metabolism, and energy balance. Although symptomatic care is the foundation of conventional therapy for many disorders, CRISPR presents a possible curative method by genome-level mutation correction. Reduced off-target effects and exact gene alterations with great specificity made possible by advances in CRISPR-Cas9, base editing, and prime editing make genome editing a feasible therapeutic alternative for many diseases [42].

Usually resulting from mutations in important genes involved in protein aggregation, mitochondrial malfunction, and synaptic integrity, neurodegenerative disorders are typified by increasing neuronal death. Widely studied as a means to change or silence pathogenic alleles, restore normal gene activity, and stop neurodegeneration is CRISPR [43].

The APOE4 allele of the Apolipoprotein E (APOE) gene raises the likelihood of amyloid plaque buildup and neural toxicity, so one of the most important genetic risk factors for Alzheimer’s disease (AD). Those with the homozygous APOE4/4 genotype have up to 15-fold higher risk of late-onset AD than those with either protective APOE2 or APOE3 alleles. Aiming to turn APOE4 into the APOE3 variation, which is linked with decreased amyloid buildup and neuroprotection, CRISPR-based genome editing techniques Using base editors, researchers have effectively changed APOE4 sequences in human neurons, therefore illustrating a possible therapeutic approach to reduce Alzheimer’s risk [44].

CAG repeat expansions in the HTT gene produce a toxic mutant huntingtin (mHTT) protein, therefore causing the monogenic neurodegenerative disease known as Huntington’s disease (HD). Expanded CAG selectively repeats have been removed using CRISPR-Cas9, hence restoring normal HTT expression and stopping neurodegeneration in HD cells and animal models. Furthermore, allele-specific CRISPR editing was developed to mute the mutant HTT allele while maintaining wild-type allele expression, therefore guaranteeing proper cellular operation [45].

Mutations in genes, including LRRK2 (Leucine-Rich Repeat Kinase 2), PINK1, and SNCA (α-synuclein), help to explain mitochondrial malfunction and dopaminergic neural loss in Parkinson’s disease (PD). To lower mutant LRRK2 variations, hence lowering neurotoxicity and enhancing neuronal survival, researchers have investigated CRISPR-based techniques. Alpha-synuclein gene expression has also been altered using CRISPR, therefore reducing the generation of harmful protein aggregates driving Parkinson’s pathogenesis. Delaying disease progression and maybe correcting neurodegeneration in patients with genetically determined PD subtypes, these CRISPR treatments have great promise [46].

### 3.5. CRISPR in Metabolic Disorders

Metabolic illnesses mostly resulting from dysregulated energy balance, insulin resistance, and abnormalities in lipid metabolism include diabetes, obesity, and dyslipidemia globally. Strong genetic foundations of these diseases make them ideal candidates for gene therapy driven by CRISPR. Among the most fascinating applications of CRISPR in metabolic disorders is editing PCSK9 (Proprotein Convertase Subtilisin/Kexin Type 9), a basic regulator of cholesterol metabolism. PCSK9 speeds down the degradation of the LDL receptor (LDLR), therefore raising LDL cholesterol levels and raising risk of cardiovascular disease (CVD). Loss-of-function mutations in PCSK9 are clearly associated with naturally low cholesterol levels and atherosclerosis prevention. CRISPR-Cas9 has changed PCSK9 expression in hepatocytes, thereby reducing LDL cholesterol and so decreasing atherosclerosis in preclinical animals. Eliminating the need for lifetime statin prescription, this approach could offer a one-time genetic therapy for hypercholesterolemia [26].

Researchers have looked into CRISPR to increase insulin sensitivity and restore pancreatic β-cell function in diabetes. Type 2 diabetes (T2D) has a feature whereby polymorphisms in genes including IRS1 (Insulin Receptor Substrate 1) and IRS2, which regulate insulin signaling routes, define insulin resistance. Insulin sensitivity in skeletal muscle and adipose tissue has been increased by means of IRS1 and IRS2 modification mediated by CRISPR, therefore enhancing glucose absorption and metabolic control [47].

Moreover, under development are pancreatic β-cell regeneration methods based on CRISPR to restore type 1 diabetes (T1D) insulin output. By means of converting pancreatic progenitor cells into insulin-producing β-cells, researchers want to offer an endogenous source of insulin free of exogenous insulin treatment or pancreatic islet transplantation using CRISPR activation [48].

Obesity, the main risk factor for metabolic syndrome, is affected by genetic variations in leptin (LEP), leptin receptor (LEP), and melanocortin 4 receptor (MC4R). Editing these genes using CRISPR has been studied to affect appetite control and energy expenditure. Using disruption of MC4R brought about by CRISPR, obesity susceptibility and potential therapy targets have been studied in mice models. Furthermore, genome editing approaches are under development to improve thermogenesis and lipid oxidation aimed at uncoupling protein 1 (UCP1) and adiponectin (ADIPOQ), offering viable therapeutic options for genetic obesity syndromes [29].

## 4. Integrating AI, CRISPR, and Pharmacogenomics for Precision Medicine

The convergence of artificial intelligence (AI), CRISPR-based genome editing, and pharmacogenomics can transform precision medicine by enabling personalized drug therapies, optimized gene-editing strategies, and data-driven therapeutic interventions. Pharmacogenomics seeks to understand how genetic variations impact drug metabolism, efficacy, and safety, allowing individualized treatment regimens. CRISPR gene-editing technology offers a method for precise genomic modifications, facilitating the correction of disease-causing mutations and the validation of pharmacogenomic variants. AI is critical in integrating these technologies by analyzing complex multi-omics datasets, predicting gene-editing outcomes, and optimizing drug-target interactions. By combining these three disciplines, researchers can develop data-driven, patient-specific interventions that improve therapeutic efficacy and reduce adverse drug reactions (ADRs) (Table 2) [4].

### 4.1. The Convergence of AI, CRISPR, and Pharmacogenomics in Precision Medicine

The synergy between AI, CRISPR, and pharmacogenomics represents a paradigm shift in biomedical research and clinical applications. Pharmacogenomics generates vast datasets from genomic sequencing, transcriptomics, proteomics, and metabolomics, providing valuable insights into genetic variants influencing drug metabolism and response. However, translating these insights into clinical decision-making and gene-editing interventions requires sophisticated computational tools. AI algorithms, particularly machine learning (ML) and deep learning (DL) have emerged as essential tools for identifying pharmacogenomic biomarkers, predicting drug responses, and refining CRISPR editing strategies [30].

CRISPR-based genome editing has provided an unprecedented ability to modify pharmacogenes, correct mutations affecting drug metabolism, and enhance therapeutic outcomes. AI is revolutionizing CRISPR applications by improving guide RNA (gRNA) design, minimizing off-target effects, and predicting DNA repair outcomes. Moreover, AI-driven models have been instrumental in personalizing CRISPR therapies by analyzing individual patient genomes to tailor gene-editing strategies. AI, CRISPR, and pharmacogenomics are being applied across multiple disease domains, including oncology, metabolic disorders, and neurodegenerative diseases, marking a new era in data-driven precision medicine [36].

### 4.2. AI-Enhanced Pharmacogenomic Analysis for Personalized Medicine

Pharmacogenomics relies on identifying genetic variants that influence drug response, efficacy, and toxicity. AI-driven computational tools have significantly improved the process of analyzing pharmacogenomic data and identifying actionable genetic markers. Machine learning algorithms can now predict patient-specific drug responses based on genomic, transcriptomic, and proteomic datasets [32].

Deep learning models, such as convolutional neural networks (CNNs) and recurrent neural networks (RNNs), have been employed to detect complex gene-drug interactions and identify previously unrecognized pharmacogenomic associations. AI-powered risk stratification models are used to predict drug-induced adverse effects, ensuring that high-risk patients receive alternative therapies or adjusted dosages. AI-assisted whole-genome sequencing (WGS) analysis has facilitated the discovery of novel pharmacogenetic interactions, providing insights into how rare genetic variants contribute to drug metabolism variability. Additionally, AI is refining polygenic risk score (PRS) models, which integrate multiple pharmacogenomic variants to predict an individual’s likelihood of responding to specific medications. These AI-driven advancements are accelerating the adoption of genetically informed prescribing practices, ensuring treatments are tailored to each patient’s genetic makeup [33].

### 4.3. AI-Optimized CRISPR Genome Editing for Precision Therapy

CRISPR has revolutionized precision medicine by enabling highly specific and efficient gene-editing capabilities. However, challenges such as off-target mutations, unpredictable repair outcomes, and variability in editing efficiency must be addressed to ensure the clinical success of CRISPR-based therapies. AI is crucial in optimizing CRISPR interventions by enhancing gRNA design, predicting off-target effects, and improving repair pathway outcomes [34].

Machine learning models trained on CRISPR screening datasets can predict which gRNA sequences will produce the most precise and effective edits while minimizing unintended genomic modifications. AI-assisted CRISPR design tools such as DeepSpCas9 and CRISPRoff have significantly improved gene-editing accuracy and specificity. Moreover, AI is being integrated into prime editing and base editing technologies to enable single-nucleotide modifications without inducing double-strand breaks (DSBs), reducing the risk of genomic instability [35].

Researchers can now develop personalized gene therapies for monogenic disorders, cancer, and rare genetic diseases by leveraging AI-powered CRISPR optimizations. AI-CRISPR integration is also explored in ex vivo gene-editing applications, such as engineering patient-derived stem cells for personalized regenerative medicine. These advancements are paving the way for safe and precise gene-editing therapies tailored to an individual’s genetic profile [36].

### 4.4. AI-Guided Drug Repurposing and CRISPR-Driven Functional Genomics

Drug repurposing, the process of identifying new therapeutic uses for existing drugs, has been significantly enhanced by AI and CRISPR functional genomics. AI-powered drug repurposing algorithms analyze vast pharmacogenomic datasets to identify existing drugs that could be repurposed for new indications. Deep learning models trained on multi-omics data, drug-target interaction networks, and clinical trial outcomes can now predict novel drug–disease associations with high accuracy [3].

CRISPR-based functional genomics complements AI-driven drug repurposing by enabling high-throughput genetic screens to identify essential genes and pathways in disease pathogenesis. CRISPR knockout and activation screens have been used to uncover synthetic lethal interactions in cancer, leading to the identification of new drug targets for precision oncology. By integrating AI-based predictive modeling with CRISPR gene-editing screens, researchers can accelerate drug discovery and develop targeted therapies for genetically defined patient populations [37].

### 4.5. AI, CRISPR, and Pharmacogenomics in Immunotherapy and Oncology

AI and CRISPR are transforming cancer immunotherapy and personalized oncology by optimizing immune checkpoint inhibition, CAR-T cell engineering, and tumor microenvironment modulation. AI-driven patient stratification models can now predict which patients will respond to immune checkpoint inhibitors (PD-1, CTLA-4 blockade), improving treatment outcomes in oncology [5].

CRISPR has revolutionized CAR-T cell therapy by enabling the precise genetic modification of T cells to enhance their anti-tumor activity. AI-powered single-cell transcriptomics and T-cell receptor (TCR) sequencing are used to identify optimal tumor antigens for CAR-T therapy, ensuring greater specificity and efficacy. Additionally, AI-assisted CRISPR knockout screens have identified key immune evasion mechanisms in tumors, leading to the developing of next-generation cancer immunotherapies [38].

### 4.6. AI-CRISPR Integration for Multi-Omics Data Interpretation in Precision Medicine

Multi-omics data integration is essential for understanding disease mechanisms, drug responses, and gene-environment interactions. AI-driven computational frameworks are being developed to integrate genomic, transcriptomic, proteomic, and metabolomic datasets, providing a comprehensive view of disease progression and treatment responses. CRISPR-based gene perturbation studies combined with AI-driven multi-omics analysis are used to identify novel therapeutic targets and validate pharmacogenomic biomarkers. AI-enhanced single-cell multi-omics analysis revealed cell-type-specific drug responses, enabling personalized treatment strategies based on individual cellular profiles [39].

## 5. Ethical, Regulatory, and Implementation Challenges

Integrating AI, CRISPR, and pharmacogenomics in precision medicine presents immense potential for revolutionizing disease diagnosis, drug development, and patient-specific therapies. However, the widespread adoption of these technologies also introduces complex ethical, regulatory, and implementation challenges that must be carefully addressed to ensure their responsible and equitable use. These challenges span from genetic modifications driven by AI, data privacy concerns in pharmacogenomic studies, and regulatory hurdles in AI-powered gene therapies to the broader issue of transitioning these technologies from research environments into clinical practice [40].

One of the primary ethical dilemmas associated with AI-driven genetic modifications is the potential for unintended consequences of genome editing. While CRISPR-based therapies hold promise for correcting disease-causing mutations, concerns arise regarding germline editing, where future generations could inherit genetic modifications. The ability of AI to predict genetic variations and optimize CRISPR gene-editing strategies raises questions about the potential misuse of genome editing for non-therapeutic enhancements, such as cognitive or physical trait modifications. Such possibilities challenge bioethical principles related to genetic equity, informed consent, and societal implications of altering the human genome. The need for strict ethical frameworks to govern AI-driven CRISPR applications is paramount, ensuring that genome editing remains confined to medically necessary interventions and does not cross into ethically contentious territories [41].

In addition to ethical concerns, privacy and security issues in AI-powered pharmacogenomics have emerged as critical challenges. Pharmacogenomic data, derived from whole-genome sequencing (WGS) and multi-omics analysis, contain highly sensitive genetic information that can reveal a person’s disease risks, ancestry, and drug response patterns. AI-driven algorithms process these data to generate predictive drug response and disease susceptibility models. Still, concerns arise regarding data breaches, genetic discrimination, and the misuse of personal genetic information. The growing adoption of AI-driven pharmacogenomics in electronic health records (EHRs) necessitates the development of secure data storage, encryption technologies, and strict patient consent protocols to prevent unauthorized access and misuse of genomic data [42].

Regulatory challenges further complicate the clinical implementation of AI-driven gene therapy and drug response prediction models. Regulatory agencies, such as the U.S. Food and Drug Administration (FDA), the European Medicines Agency (EMA), and the National Institutes of Health (NIH), are actively developing guidelines to govern AI-based pharmacogenomic analysis, CRISPR gene-editing applications, and AI-powered drug discovery platforms. However, AI models’ dynamic and evolving nature presents a challenge to conventional regulatory frameworks, which are often designed for static, well-defined interventions rather than continuously learning AI systems. The lack of standardized validation protocols for AI-generated pharmacogenomic predictions raises concerns regarding AI-driven recommendations’ clinical reliability and reproducibility. Furthermore, ensuring transparency in AI decision-making processes is crucial, as physicians and regulatory bodies must be able to interpret and validate AI-generated results before incorporating them into patient care [43].

Beyond regulatory concerns, bridging the gap between research and real-world clinical applications remains a significant hurdle. Despite promising advances in AI-powered pharmacogenomics and CRISPR-based genome editing, the transition from experimental models to routine clinical use is often slowed by limited access to high-quality datasets, the high cost of implementing AI-driven tools, and the need for extensive clinical validation. Many AI-driven pharmacogenomic models are trained on population-specific genomic datasets, raising concerns about their generalizability across diverse ethnic backgrounds. Addressing this issue requires global collaborative efforts to build diverse genomic databases that ensure AI-driven pharmacogenomic tools apply to all populations, reducing health disparities in precision medicine [44].

## 6. Future Directions

The future of AI-powered pharmacogenomics and genome editing is poised to bring groundbreaking advancements in precision medicine, with emerging trends shaping the next decade of biomedical research and clinical applications. One of the most promising directions is the evolution of explainable AI (XAI), which aims to make AI-driven predictions transparent, interpretable, and clinically actionable. Traditional deep learning models function as black-box algorithms, making understanding how AI reaches a specific pharmacogenomic or gene-editing recommendation difficult. XAI seeks to bridge this gap by providing interpretable outputs, enabling physicians and researchers to validate AI-generated findings before making clinical decisions. In AI-CRISPR integration, explainability is crucial in ensuring that gene-editing interventions are accurately targeted with minimal off-target effects, enhancing the safety and reliability of CRISPR-based therapeutics [45].

Another emerging trend is the integration of AI with synthetic biology and high-throughput CRISPR screens, accelerating the discovery of new druggable targets and therapeutic interventions. AI-powered functional genomics studies uncover previously unrecognized gene-drug interactions, leading to novel drug repurposing opportunities. AI-driven multi-omics analysis enables researchers to construct highly personalized treatment strategies, incorporating genomic, transcriptomic, proteomic, and metabolomic data to tailor therapies to individual patients with unprecedented precision [3].

In oncology, the future implications of AI-enhanced genome editing extend beyond pharmacogenomics to personalized cancer treatment strategies. AI-driven CRISPR-based tumor profiling is being developed to predict tumor evolution, identify resistance mechanisms, and optimize combination therapies for cancer patients. AI also enables real-time monitoring of immune checkpoint inhibitor responses, improving patient selection for immunotherapy treatments. These AI-powered approaches are expected to revolutionize personalized cancer care by ensuring that each patient receives the most effective, genetically tailored treatment regimen [46].

Beyond cancer, AI-powered genome engineering opens new possibilities for treating rare genetic disorders that currently lack effective therapies. AI-enhanced prime editing and base editing are emerging as highly precise alternatives to conventional CRISPR-Cas9 editing, allowing for error-free single-nucleotide corrections without inducing double-strand breaks. These AI-driven gene-editing tools are being investigated for in vivo delivery using nanoparticle-based CRISPR systems, expanding the therapeutic potential of genome editing beyond ex vivo applications. Further, to ensure the benefits of AI-powered pharmacogenomics and genome editing would be equitably distributed. The future efforts of genome engineering must prioritize health equity and accessibility, particularly for underserved and marginalized populations. Integrating AI with frameworks that address social determinants of health could help reduce disparities in access to precision medicine treatment and innovation. Therefore, the combination of AI-guided delivery systems, precision genome editing, and pharmacogenomic profiling could lead to the development of one-time, curative gene therapies for rare monogenic disorders [47,48,49].

## 7. Conclusions

Integrating AI, CRISPR, and pharmacogenomics has set the stage for the next generation of precision medicine, offering unparalleled opportunities for personalized therapy, drug discovery, and gene-based interventions. AI-driven advancements enhance the accuracy of pharmacogenomic predictions, optimize CRISPR-based genome editing, and accelerate the identification of novel therapeutic targets. However, implementing these technologies must be approached with caution and responsibility, addressing ethical, regulatory, and data privacy concerns while ensuring transparency, clinical validation, and equitable access.

As AI continues to evolve, its role in interpreting multi-omics data, guiding gene-editing strategies, and personalizing treatment regimens will further redefine modern healthcare. Developing explainable AI models, AI-driven synthetic biology approaches, and genome-wide CRISPR functional studies will provide deeper insights into disease mechanisms and therapeutic interventions. With continued advancements and regulatory refinements, AI-powered precision medicine will ultimately revolutionize how diseases are diagnosed, treated, and prevented, ensuring a future where healthcare is truly personalized, predictive, and patient-centered.

## Figures and Tables

**Table 1:** Comparison of the CRISPR-Cas9, CRISPR-Cas12, and CRISPR-Cas13 systems.

Feature	CRISPR-Cas9	CRISPR-Cas12	CRISPR-Cas13
Target Type	Double-stranded DNA	Single-stranded and double-stranded DNA	Single-stranded RNA
Cleavage Mechanism	Creates double-strand breaks (DSBs)	Creates single-strand staggered cuts	Cleave RNA molecules
Guide RNA (gRNA)	Single guide RNA (sgRNA)	Single guide RNA (sgRNA)	CRISPR RNA (crRNA)
Recognition Motif (PAM or PFS)	PAM sequence (5′-NGG-3′)	PAM sequence (5′-TTTV-3′)	PFS (Protospacer Flanking Site) instead of PAM
Editing Precision	High, but off-target effects possible	Higher specificity than Cas9	High specificity for RNA editing
Primary Applications	Gene knockout, gene correction, genome-wide screening	Genome editing, diagnostics (e.g., SHERLOCK)	RNA interference, viral RNA targeting, transcriptome regulation
Advantages	Efficient for genome editing, widely studied, well-characterized	Higher specificity, useful for diagnostics and collateral activity, enables detection applications	Can target RNA without affecting genome, potential for antiviral therapies
Limitations	Potential for off-target mutations requires PAM sequence	Less well-characterized than Cas9, requires PAM sequence	Collateral RNA degradation limits specificity and is not useful for DNA editing

**Table 2:** Integration of AI, CRISPR, and pharmacogenomics in precision medicine, highlighting key applications, methodologies, and challenges across various therapeutic domains.

AI, CRISPR, and Pharmacogenomics Concept	Key Focus	Applications	AI Techniques Used	Challenges
Drug Repurposing and Functional Genomics	AI-powered drug repurposing and CRISPR functional genomics to identify new therapeutic applications of existing drugs and novel drug targets.	Oncology, rare diseases, metabolic disorders, identification of synthetic lethal interactions.	Neural networks, AI-driven drug-target interaction prediction, deep learning-based functional genomics screening.	Validation of AI-identified drug repurposing candidates, ethical considerations in genetic screening.
Immunotherapy and Oncology	Optimization of cancer immunotherapy through AI-driven patient stratification, CRISPR-enhanced CAR-T cell therapy, and immune checkpoint modulation.	Tumor microenvironment analysis, AI-powered immune profiling, CRISPR-engineered immunotherapies.	AI-based single-cell sequencing analysis, AI-guided CRISPR knockout studies, immune response prediction.	Patient-specific immunotherapy optimization, overcoming tumor immune evasion mechanisms.
Multi-Omics Data Interpretation	AI-driven multi-omics data integration for understanding disease mechanisms, drug responses, and personalized treatment strategies.	Personalized multi-omics analysis, single-cell transcriptomics, and disease pathway modeling.	Integrating AI with genomics, transcriptomics, proteomics, and metabolomics data analysis.	Standardization of multi-omics data interpretation, regulatory challenges in AI-driven precision medicine.
Neurodegenerative Disease Therapy	CRISPR-based correction of neurodegenerative disease mutations, AI-guided identification of genetic targets for personalized therapy.	Alzheimer’s, Parkinson’s, Huntington’s disease, CRISPR-based neuroprotection.	Machine learning-based biomarker discovery, AI-guided CRISPR target selection, and deep learning for disease progression modeling.	Ethical concerns in CRISPR neuro-editing, ensuring AI accuracy in neurogenomic predictions.
Metabolic Disorder Management	Precision genome editing for diabetes, obesity, and cholesterol management, AI-driven analysis of metabolic pathways.	CRISPR-driven insulin sensitivity modulation, lipid metabolism optimization, personalized treatment for metabolic disorders.	AI-enhanced metabolic profiling, deep learning for CRISPR-mediated genetic corrections.	Long-term safety of CRISPR-based metabolic modifications, AI-driven identification of unintended gene interactions.
AI-Driven Pharmacovigilance	AI-driven real-time monitoring of adverse drug reactions (ADRs), CRISPR validation of pharmacogenomic safety markers.	Early detection of ADRs, pharmacogenomic safety monitoring, and AI-enhanced drug safety assessments.	Natural language processing (NLP) for pharmacovigilance data, AI-assisted safety screening of CRISPR modifications.	Regulatory approval of AI-powered pharmacovigilance systems, balancing AI automation with clinical oversight.
Synthetic Biology and AI-CRISPR Integration	AI-powered synthetic biology tools for CRISPR-based genome engineering, designing gene circuits for therapeutic applications.	AI-designed gene circuits for cell therapy, CRISPR-driven biosynthetic pathway modifications.	AI-assisted protein engineering, machine learning-driven pathway modeling, CRISPR gene circuit optimization.	Ethical concerns in AI-driven synthetic biology, potential risks of unintended gene expression alterations.
Gene Therapy Optimization	Improving gene therapy efficiency using AI-optimized CRISPR delivery mechanisms and patient-specific therapeutic modeling.	Rare genetic disease therapy, AI-enhanced vector selection for CRISPR delivery, CRISPR-based ex vivo gene correction.	AI-powered vector design, deep learning for optimizing CRISPR efficiency, AI-driven prediction of therapeutic outcomes.	Minimizing CRISPR off-target effects in gene therapy, regulatory challenges in AI-assisted gene therapy development.
Personalized Regenerative Medicine	AI-CRISPR-engineered stem cells for regenerative medicine developed personalized tissue and organ repair strategies.	Tissue regeneration, CRISPR-based iPSC therapies, AI-powered stem cell differentiation modeling.	AI-driven stem cell fate prediction, machine learning-guided tissue engineering, CRISPR-based genome stability analysis.	AI-driven stem cell differentiation reliability, CRISPR safety in regenerative medicine, patient-specific variability.

## Data Availability

All relevant data are contained within this manuscript. There are no additional datasets associated with this study.

## Abbreviations

The following abbreviations are used in this manuscript:

AI	Artificial Intelligence
CRISPR	Clustered Regularly Interspaced Short Palindromic Repeats
PGx	Pharmacogenomics
CAR-T	Chimeric Antigen Receptor T-cell
NLP	Natural Language Processing
GWAS	Genome-Wide Association Study
XAI	Explainable AI
TMB	Tumor Mutational Burden
miRNA	microRNA
lncRNA	Long Non-Coding RNA
HDR	Homology-Directed Repair
NHEJ	Non-Homologous End Joining
iPSC	Induced Pluripotent Stem Cells
TCR	T-Cell Receptor
FDA	Food and Drug Administration
TP53	Tumor Protein p53
CYP	Cytochrome P450
HLA	Human Leukocyte Antigen
NGS	Next-Generation Sequencing
PAM	Protospacer Adjacent Motif

## References

[1] Marques L., Costa B., Pereira M., Silva A., Santos J., Saldanha L., Silva I., Magalhães P., Schmidt S., Vale N. (2024). Advancing Precision Medicine: A Review of Innovative In Silico Approaches for Drug Development, Clinical Pharmacology and Personalized Healthcare. Pharmaceutics.

[2] Krzyszczyk P., Acevedo A., Davidoff E.J., Timmins L.M., Marrero-Berrios I., Patel M., White C., Lowe C., Sherba J.J., Hartmanshenn C. (2018). The growing role of precision and personalized medicine for cancer treatment. Technology.

[3] Serrano D.R., Luciano F.C., Anaya B.J., Ongoren B., Kara A., Molina G., Ramirez B.I., Sánchez-Guirales S.A., Simon J.A., Tomietto G. (2024). Artificial Intelligence (AI) Applications in Drug Discovery and Drug Delivery: Revolutionizing Personalized Medicine. Pharmaceutics.

[4] Ho D., Quake S.R., McCabe E.R.B., Chng W.J., Chow E.K., Ding X., Gelb B.D., Ginsburg G.S., Hassenstab J., Ho C.M. (2020). Enabling Technologies for Personalized and Precision Medicine. Trends Biotechnol..

[5] Bhat A.A., Nisar S., Mukherjee S., Saha N., Yarravarapu N., Lone S.N., Masoodi T., Chauhan R., Maacha S., Bagga P. (2022). Integration of CRISPR/Cas9 with artificial intelligence for improved cancer therapeutics. J. Transl. Med..

[6] Taherdoost H., Ghofrani A. (2024). AI’s role in revolutionizing personalized medicine by reshaping pharmacogenomics and drug therapy. Intell. Pharm..

[7] Ahmed S., Zhou Z., Zhou J., Chen S.Q. (2016). Pharmacogenomics of Drug Metabolizing Enzymes and Transporters: Relevance to Precision Medicine. Genom. Proteom. Bioinform..

[8] Nahid N.A., Johnson J.A. (2022). CYP2D6 pharmacogenetics and phenoconversion in personalized medicine. Expert Opin. Drug Metab. Toxicol..

[9] Sissung T.M., Goey A.K., Ley A.M., Strope J.D., Figg W.D. (2014). Pharmacogenetics of membrane transporters: A review of current approaches. Methods Mol. Biol..

[10] Principi N., Petropulacos K., Esposito S. (2023). Impact of Pharmacogenomics in Clinical Practice. Pharmaceuticals.

[11] Adithan C., Subathra A. (2016). NAT2 gene polymorphism: Covert drug interaction causing phenytoin toxicity. Indian J. Med. Res..

[12] Urbančič D., Jukič M., Šmid A., Gobec S., Jazbec J., Mlinarič-Raščan I. (2025). Thiopurine S-methyltransferase—An important intersection of drug-drug interactions in thiopurine treatment. Biomed. Pharmacother..

[13] Unissa A.N., Sukumar S., Hanna L.E. (2017). The Role of N-Acetyl Transferases on Isoniazid Resistance from Mycobacterium tuberculosis and Human: An In Silico Approach. Tuberc. Respir. Dis..

[14] Roden D.M., Wilke R.A., Kroemer H.K., Stein C.M. (2011). Pharmacogenomics: The genetics of variable drug responses. Circulation.

[15] Ran F.A., Hsu P.D., Wright J., Agarwala V., Scott D.A., Zhang F. (2013). Genome engineering using the CRISPR-Cas9 system. Nat. Protoc..

[16] Liu W., Li L., Jiang J., Wu M., Lin P. (2021). Applications and challenges of CRISPR-Cas gene-editing to disease treatment in clinics. Precis. Clin. Med..

[17] Aljabali A.A.A., El-Tanani M., Tambuwala M.M. (2024). Principles of CRISPR-Cas9 technology: Advancements in genome editing and emerging trends in drug delivery. J. Drug Deliv. Sci. Technol..

[18] Xue C., Greene E.C. (2021). DNA Repair Pathway Choices in CRISPR-Cas9-Mediated Genome Editing. Trends Genet..

[19] Shaikh T.H. (2017). Copy Number Variation Disorders. Curr. Genet. Med. Rep..

[20] Bhatt D.K., Basit A., Zhang H., Gaedigk A., Lee S.B., Claw K.G., Mehrotra A., Chaudhry A.S., Pearce R.E., Gaedigk R. (2018). Hepatic Abundance and Activity of Androgen- and Drug-Metabolizing Enzyme UGT2B17 Are Associated with Genotype, Age, and Sex. Drug Metab. Dispos..

[21] Loureiro A., da Silva G.J. (2019). CRISPR-Cas: Converting A Bacterial Defence Mechanism into A State-of-the-Art Genetic Manipulation Tool. Antibiotics.

[22] Asmamaw M., Zawdie B. (2021). Mechanism and Applications of CRISPR/Cas-9-Mediated Genome Editing. Biologics.

[23] Sioson V.A., Kim M., Joo J. (2021). Challenges in delivery systems for CRISPR-based genome editing and opportunities of nanomedicine. Biomed. Eng. Lett..

[24] Alamillo J.M., López C.M., Martínez Rivas F.J., Torralbo F., Bulut M., Alseekh S. (2023). Clustered regularly interspaced short palindromic repeats/CRISPR-associated protein and hairy roots: A perfect match for gene functional analysis and crop improvement. Curr. Opin. Biotechnol..

[25] Xu C.L., Ruan M.Z.C., Mahajan V.B., Tsang S.H. (2019). Viral Delivery Systems for CRISPR. Viruses.

[26] Yoon H., Shaw J.L., Haigis M.C., Greka A. (2021). Lipid metabolism in sickness and in health: Emerging regulators of lipotoxicity. Mol. Cell.

[27] Tsuchida C.A., Wasko K.M., Hamilton J.R., Doudna J.A. (2024). Targeted nonviral delivery of genome editors in vivo. Proc. Natl. Acad. Sci. USA.

[28] Mehta A., Merkel O.M. (2020). Immunogenicity of Cas9 Protein. J. Pharm. Sci..

[29] Saeed S., Bonnefond A., Froguel P. (2025). Obesity: Exploring its connection to brain function through genetic and genomic perspectives. Mol. Psychiatry.

[30] Molla G., Bitew M. (2024). Revolutionizing Personalized Medicine: Synergy with Multi-Omics Data Generation, Main Hurdles, and Future Perspectives. Biomedicines.

[31] Chakravarthi B.V., Nepal S., Varambally S. (2016). Genomic and Epigenomic Alterations in Cancer. Am. J. Pathol..

[32] Kalinin A.A., Higgins G.A., Reamaroon N., Soroushmehr S., Allyn-Feuer A., Dinov I.D., Najarian K., Athey B.D. (2018). Deep learning in pharmacogenomics: From gene regulation to patient stratification. Pharmacogenomics.

[33] Askr H., Elgeldawi E., Aboul Ella H., Elshaier Y.A.M.M., Gomaa M.M., Hassanien A.E. (2023). Deep learning in drug discovery: An integrative review and future challenges. Artif. Intell. Rev..

[34] Guo C., Ma X., Gao F., Guo Y. (2023). Off-target effects in CRISPR/Cas9 gene editing. Front. Bioeng. Biotechnol..

[35] Erdoğan S. (2024). Integration of Artificial Intelligence and Genome Editing System for Determining the Treatment of Genetic Disorders. Balk. Med. J..

[36] Chehelgerdi M., Chehelgerdi M., Khorramian-Ghahfarokhi M., Shafieizadeh M., Mahmoudi E., Eskandari F., Rashidi M., Arshi A., Mokhtari-Farsani A. (2024). Comprehensive review of CRISPR-based gene editing: Mechanisms, challenges, and applications in cancer therapy. Mol. Cancer.

[37] Bock C., Datlinger P., Chardon F., Coelho M.A., Dong M.B., Lawson K.A., Lu T., Maroc L., Norman T.M., Song B. (2022). High-content CRISPR screening. Nat. Rev. Methods Primers.

[38] Tao R., Han X., Bai X., Yu J., Ma Y., Chen W., Zhang D., Li Z. (2024). Revolutionizing cancer treatment: Enhancing CAR-T cell therapy with CRISPR/Cas9 gene editing technology. Front. Immunol..

[39] Subramanian I., Verma S., Kumar S., Jere A., Anamika K. (2020). Multi-omics Data Integration, Interpretation, and Its Application. Bioinform. Biol. Insights.

[40] Jiang W., Oikonomou P., Tavazoie S. (2020). Comprehensive Genome-wide Perturbations via CRISPR Adaptation Reveal Complex Genetics of Antibiotic Sensitivity. Cell.

[41] Ayanoğlu F.B., Elçin A.E., Elçin Y.M. (2020). Bioethical issues in genome editing by CRISPR-Cas9 technology. Turk. J. Biol..

[42] Gershon E.S., Alliey-Rodriguez N., Grennan K. (2014). Ethical and public policy challenges for pharmacogenomics. Dialogues Clin. Neurosci..

[43] Minssen T., Gerke S., Aboy M., Price N., Cohen G. (2020). Regulatory responses to medical machine learning. J. Law Biosci..

[44] Lafi Z., Ata T., Asha S. (2025). CRISPR in clinical diagnostics: Bridging the gap between research and practice. Bioanalysis.

[45] Johnson K.B., Wei W.Q., Weeraratne D., Frisse M.E., Misulis K., Rhee K., Zhao J., Snowdon J.L. (2021). Precision Medicine, AI, and the Future of Personalized Health Care. Clin. Transl. Sci..

[46] Bhinder B., Gilvary C., Madhukar N.S., Elemento O. (2021). Artificial Intelligence in Cancer Research and Precision Medicine. Cancer Discov..

[47] Abdalla M.M.I. (2024). Advancing diabetes management: Exploring pancreatic beta-cell restoration’s potential and challenges. World J. Gastroenterol..

[48] Karpov D.S., Sosnovtseva A.O., Pylina S.V., Bastrich A.N., Petrova D.A., Kovalev M.A., Shuvalova A.I., Eremkina A.K., Mokrysheva N.G. (2023). Challenges of CRISPR/Cas-Based Cell Therapy for Type 1 Diabetes: How Not to Engineer a “Trojan Horse”. Int. J. Mol. Sci..

[49] Dixit S., Kumar A., Srinivasan K., Vincent P., Ramu Krishnan N. (2023). Advancing genome editing with artificial intelligence: Opportunities, challenges, and future directions. Front. Bioeng. Biotechnol..

